# New Directions in Police Academy Training: A Call to Action †

**DOI:** 10.3390/ijerph16244941

**Published:** 2019-12-06

**Authors:** Daniel M. Blumberg, Michael D. Schlosser, Konstantinos Papazoglou, Sarah Creighton, Chief Chuck Kaye

**Affiliations:** 1 California School of Professional Psychology, Alliant International University, San Diego, CA 92131, USA; 2 Police Training Institute, University of Illinois at Urbana-Champaign, Champaign, IL 61820, USA; schlossr@illinois.edu; 3 Yale School of Medicine, New Haven, CT 06511, USA; konstantinos.papazoglou@yale.edu; 4 San Diego Police Department, San Diego, CA 92101, USA; stcr8n@gmail.com; 5 Coronado Police Department, Coronado, CA 92118, USA

**Keywords:** police training, psychological skills, wellness, community relations

## Abstract

The complexities of modern policing require law enforcement agencies to expand how officers are trained to do their jobs. It is not sufficient for training to focus solely on the law or on perishable skills; such as arrest and control; defensive tactics; driving; and firearms. The present manuscript addresses the critical importance of infusing academy training with the psychological skills essential for officers to meet the contemporary challenges of police work. The authors suggest that the skills (i.e., cognitive; emotional; social; and moral) discussed in this paper may improve officers’ wellness as well as promote relationships between police officers and community members. Specific methods of incorporating these skills in academy training are offered.

## 1. Introduction

Effective policing has always required a unique combination of physical, cognitive, emotional, and interpersonal skills. Although the majority of an officer’s time spent on duty involves non-threatening duties such as responding to a traffic accident, it is necessary to prepare officers for the worse-case scenarios. According to the International Association of Chiefs of Police (2001), police officers’ use of force rate is just 3.61 per 10,000 service calls [1]. However, in those rare situations in which an officer is called upon to use force, these learned skills and tactics become critical. This can lead academy directors and trainers to emphasize skills such as firearms and defensive tactics during academy training. The present paper highlights the importance of other skills that should receive greater attention during academy training.

Today’s police officers carry more tools on their equipment belts (e.g., Tasers) and bodies (e.g., body-worn cameras), utilize more equipment in their patrol cars (e.g., computers), and face more public scrutiny of their actions due to smart phones and social media than officers from prior generations. It can be argued that the job has never been more demanding or, for that matter, more stressful. In the least, there is little dispute that contemporary policing is extremely complex and challenging. For this reason, law enforcement agencies are obligated to hire, train, and retain a cadre of the most psychologically fit police officers.

The primary areas of training for police academies fall into the following five categories: operations (average hours of training = 213 h), firearms, self-defense, and use of force (168 h), self-improvement (89 h), legal education (86 h), and mental illness (10 h; [2]). In the category of self-improvement, more than half of the curriculum focuses on health and fitness. The remainder of self-improvement training consists primarily of ethics and integrity, communications, professionalism, and stress prevention/management [2]. Eighty-one percent of academies provide stress prevention/management training, with an average time of six hours [2]. However, police academy training is not standardized across the United States. The curricula in academies vary by state and often even by academy within a state.

Police training is responsible for preparing new hires for this difficult career. Officers attend a relatively brief police academy, which is followed by training in the field. The U.S. Department of Justice published an analysis of the nation’s “664 state and local law enforcement academies” [2] (p. 1), which provides a good overview of the content of academy training. There are various academy formats, including full-time training for six months, part-time training for 12 months, and residential academies, which are quite similar to military boot camps. Although academy training varies greatly state to state, the average length of such training programs in the United States is 840 h [2]. Although the content of police academy training is typically mandated by state or federal governing bodies, the majority of police academies in the United States continue to train recruits using a quasi-military/boot camp approach. According to the US Bureau of Justice, quasi-military or stress-based training involving intense psychological and physical demands are still implemented in almost half of police academies nationally [2]. Another 33% of police recruits are trained in a balance of stress and non-stress environments and only 18% are trained in an academy that promotes a lower stress (less militaristic) approach to training [2]. Successful completion of the academy certifies officers with police powers in the given jurisdiction. 

Police academy training has two general aspects. The academic component takes place in classroom settings and requires recruits (or, in some academies, “cadets”) to learn the basics of law, procedures, radio codes, penal codes, etc. In California, for example, recruits spend a minimum of 664 h learning content from 42 separate learning domains [3]. This is done in a didactic format and involves formal testing in which recruits must pass each exam with a certain minimum score. The other component of the police academy involves hands-on training and includes rehearsal and scenario-based, performance appraisals in areas, which include arrest and control, defensive tactics, use of weapons, and driving. Some of these skills, such as driving, tactical firearms, and arrest and control, are considered perishable and require incumbent officers to receive periodic refresher training throughout their careers (e.g., [4]). Similar to the academic portion, recruits must demonstrate proficiency in these skills or fail that learning domain. Most academies allow recruits to fail a certain number of domains and to remediate. If any domain is not satisfactorily passed, the recruit is terminated from the academy.

Moreover, previous research has shown that a combination of lectures and theoretical classroom discussions along with practical application of theoretical knowledge into a simulated training environment has been proven to be quite efficient in improving learning, health promotion, job performance, and officers’ capacity to translate theoretical knowledge into police practice. For instance, such training programs were developed for experienced police trainers to help them incorporate resilience promotion techniques in their police training curricula with police trainees [5,6]. Analogously, effective results have been found in previous studies with special weapons and tactics (SWAT) teams where SWAT officers were taught about resilience promotion in both a classroom learning environment and a simulated reality training environment; to this end, SWAT officers who incorporated the learning materials into both classroom and simulated reality environments showed a substantially improved capacity to manage challenges on both realistic training environment as well as real life police work [7,8,9,10,11,12,13]. Therefore, a combination of classroom learning (either in-class and/or online) along with transference of classroom discussions into a realistic training venue appears to be an imperative combination for helping officers incorporate theoretical knowledge in their practical training and real life police work. 

Traditionally, police academies have been conducted in a paramilitary fashion. This means that recruits’ are held to a high standard of discipline, deportment, and regimentation while learning how to become a police officer. Often, academy training staff would be indistinguishable from military drill sergeants, who verbally harass and, even demean recruits who are not measuring up. Pushups, extra running, and writing reports are used as punishment. Although this training format builds camaraderie and a high level of esprit de corps, it tends to have a fairly high dropout rate, which may not trouble purists who are of the belief that “if they can’t cut it here, they’d never survive on the streets.” Although there is some truth to this (i.e., training should prepare recruits for the harshest conditions that they will face on the job), this format also lacks attention to individual differences in learning styles, personalities, and pre-existing interpersonal skills. 

This is not a new concern. For over 30 years, there has been a steady stream of criticism, which underscores deficiencies in police academy training when it comes to adequately preparing recruits for the actual demands of the job (e.g., [14,15]). Much of this criticism has come from officers themselves when asked to reflect on the relevance of their academy experience (e.g., [16,17]) and from police administrators who do not believe that police academy training is sufficient (e.g., [18]). The general disconnect between academy training and job preparation tends to revolve around two interrelated issues concerning the content and the delivery of the academy curriculum. The typical paramilitary format fails to prepare recruits to work in a manner consistent with the community-oriented police services model (COPS) and neglects basic principles of adult-learning theory. Essentially, in order to produce officers who are able to successfully perform community-oriented policing techniques (e.g., proactive collaboration with community members [19]), police academies must train recruits to be independent, creative problem solvers (e.g., [20,21,22,23]).

Past literature has discussed the importance for police academies to adopt an adult-learning theory model (e.g., [24]). Fundamentally, beyond the issues surrounding the best training techniques to prepare recruits to work within a community-oriented policing model, law enforcement agencies are faced with the broader question of what type of police officers are they training? Recruits who are trained in a manner that is consistent with adult-learning theory are encouraged to develop critical thinking skills, effective communication, and better emotional intelligence. However, this is generally not how police recruits are trained in practice (e.g., [23]). Moreover, academies that embrace an adult-learning model recognize the significance of how training is delivered. Specifically, academy training staff serve as strong role models who socialize officers into the agency’s culture (e.g., [16,25]). Vodde (2011) stated this quite starkly: “Thus, if recruits are constantly exposed to an autocratic, prescriptive, and discipline-oriented instructor, as opposed to one that is benevolent, fair-minded, and mentoring, such behaviors will inevitably be modeled” [26] (p. 35). Stoughton (2015) goes even further by contending that traditional training models teach officers to be afraid, which “…inevitably affects the way that officers interact with civilians” [27] (p. 228).

Keeping pace with the changing demands of contemporary policing, academy training has had to evolve. It is no longer sufficient for training to teach just the law or to focus only on the perishable skills mentioned above. It is extremely counterproductive to train recruits in an authoritarian, pedagogical format. Nevertheless, the voices calling for change in police academy practices tend to lack prescriptive details on how to accomplish this change and, more precisely, how to teach, strengthen, reinforce, and support the skills needed to graduate officers who are psychologically prepared to competently perform in the field. For example, although the Law Enforcement Foundation in Ohio identified twelve job competencies in 2001, police academies appear to have made little real progress in training recruits in many of these important skills: 

High moral/ethical standards; unbiased and understanding of diversity; service orientation; team orientation; good oral communication and listening skills; good written; communication skills; high levels of motivation, strong decision-making and problem-solving skills; good human relations skills, self-control and discipline; good planning and organization skills; and, a performance-driven attitude [14] (p. 360). 

Similarly, in 2004, the California Commission on Peace Officer Standards and Training identified ten psychological screening dimensions for agencies to consider when hiring police officers. In this manuscript, the focus is on the ways in which police academies can infuse training with these psychological skills, which recruits need in order to meet the challenges faced by today’s police officers. For convenience and clarity, these skills will be separated into four groups: cognitive, emotional, social, and moral. In addition to defining the skills, attention is paid to the ways in which specific academy experiences can teach and strengthen each skill.

## 2. Cognitive Skills

Police work is mentally challenging. This has led to a somewhat longstanding debate about the extent to which law enforcement agencies should set minimum education requirements for their new hires. The research findings, however, have been somewhat mixed. The general argument is that formal education and the experience of attending college instills in future police officers a level of mental flexibility and other psychological skills, which are not found in their peers who have only completed high school (e.g., [18,28]). More specifically, Paoline and Terrill (2007) found that, although officers with any college experience tended to use less verbal force, it was only officers who completed four years of college who used less physical force than their less educated peers [29] (p. 192). However, additional research has focused on the specific nature of officers’ formal education and found that “criminal justice students demonstrated higher levels of authoritarianism than graduates of other disciplines” [28] (p. 289). 

The discussion of formal education is contrasted with the role of experience and where that experience can be attained. For example, officers without formal education, but who have more job experience showed less use of verbal and physical force than newer officers [29] (p. 193). Therefore, it appears that formal education is not the key factor associated with police officers’ effectiveness. It is more likely that important skills, which are most often acquired during college, lead to officers’ effectiveness and are the reason why many champion a college education for police recruits. However, not all who attend college acquire these skills, and college is not the only way for individuals to obtain them. Furthermore, leaving such important skill acquisition to chance is unnecessary when police agencies can ensure that all recruits develop these skills during academy training.

Police academies can seamlessly integrate essential cognitive skill training into current academy curricula. From the Peace Officer Psychological Screening Manual, the skills in question are: 

*Decision-Making/Judgment* “involves common sense, ‘street smarts,’ and the ability to make sound decisions, demonstrated by the ability to size up situations quickly to determine and take appropriate action. This skill also involves the ability to sift through information to glean that which is important, and, once identified, to use that information effectively” [30] (p. 67).

*Impulse Control/Attention to Safety* “involves taking proper precautions and avoiding impulsive and/or unnecessarily risky behavior to ensure the safety of oneself and others. It includes the ability and inclination to think before acting—to keep one’s impetuous, knee-jerk reactions in check, and instead behave in conscious regard for the larger situation at hand” [30] (p. 59).

*Conscientiousness/Dependability* “involves diligent, reliable, conscientious work patterns and performing in a timely, logical manner in accordance with rules, regulations and organizational policies” [21] (p. 56). 

*Adaptability/Flexibility* “involves the ability to change gears and easily adjust to the many different, sudden, and sometimes competing demands of the job” [30] (p. 55).

Although current practices tend to do a better job addressing these cognitive skills than, say, the emotional skills, this is usually done indirectly. That is, acquisition of these skills is often inferred from recruits’ performance on various other assessments or evaluated in conjunction with another assessment. For example, during an evaluation of recruits’ arrest and control skills, training staff determine recruits’ ability to properly escalate use of force according to the use of force continuum (e.g., [25]). Their performance may lead to inferred conclusions about decision-making, judgment, and impulse control. There are two problems when staff make such conclusions. First, it is possible to confuse a learning deficiency with a decision-making problem; the recruit’s poor performance on the assessment may be due to a lack of understanding of or acuity with the use of force continuum rather than due to deficits in decision-making or judgment. Second, training staff members are not basing such conclusions on a direct assessment of decision-making. The importance of these cognitive skills for effective police performance dictates a need for standardized and specific measurements of the cognitive skills themselves. 

Police academies need to improve the way in which they develop recruits’ independent, critical thinking. To impart and strengthen the four cognitive skills, academies need to find a way to reduce the didactic, micromanagement of recruits (i.e., telling them exactly what to do at all times) and increase opportunities for recruits’ autonomous decision-making. This can be done in various learning domains as a normal course of action. Specifically, recruits should, without Training Officers’ presence or involvement, be given time in break-out groups to argue pros and cons of various actions. Then, after presenting the small groups’ ideas to the whole group, the recruits should be guided to examine the reasoning behind their decisions. This should also be done, after each practical exercise where recruits are required to discuss why they chose to do what they did. By hearing the rationale of peers, rather than the wrath of instructors, they will develop better critical thinking skills and improve their impulse control and decision-making.

Conscientiousness and Adaptability are not cultivated in a traditional paramilitary police academy. When the academy shifts to an adult learning model, recruits are confronted through scenarios associated with various learning domains with a requirement to be more flexible, to think on their feet, and to demonstrate conscientious work behavior. Punishment is replaced with logical consequences. For example, being late or forgetting a piece of equipment is not met with extra push-ups or having to write a report. Instead, these behaviors will result in the same outcomes a recruits will experience when such behavior happens on the job (i.e., progressive discipline). In other words, the traditional academy model does not teach recruits the importance of dependability and flexibility; it just reinforces compliance. Therefore, to build these cognitive skills, academies should mirror the supervision and discipline model of the agencies where their recruits will soon work. 

## 3. Emotional Skills

Police work is emotionally challenging. On many levels, police officers’ emotions affect how well they do their job and how long they will be able to do their job well. In terms of longevity, much has been written about the emotional toll that police work takes on officers, which can lead to, among other consequences, debilitating levels of burnout (e.g., [31,32]). A contributing factor, beyond the routine exposure to trauma and human suffering, was found to be the emotional exhaustion officers experience from constantly showing the public emotions other than what they actually feel, e.g., remaining calmly stoic when disgusted, or smiling when actually angry [33,34,35]. However, a recent study found that newer officers who had not yet been exposed to on-duty traumatic incidents were less emotionally well-adjusted than trauma-exposed senior officers [36], who have developed more resiliency. Of course, in a given moment, police officers’ acute emotions significantly impact their job performance. For example, “When they were anxious, the officers had a stronger expectation of threat, which caused them to shoot earlier and make more mistakes” [37] (p. 832).

Emotional skills can improve the extent to which police officers successfully manage the emotional challenges of the job. One such skill, according to the Peace Officer Psychological Screening Manual, is *Emotional Regulation/Stress Tolerance*, which: “involves the ability to maintain composure and stay in control, particularly during time-critical emergency events and other stressful situations. It includes taking the negative aspects of the job in stride and maintaining an even temperament, as well as accepting criticism rather than becoming overly defensive or allowing it to hamper job performance” [30] (p. 64). 

On a very promising and apropos note, research has demonstrated that police officers can be taught to improve their emotion regulation skills (e.g., [38]). Nelis and colleagues (2011) demonstrated specific and lasting improvement in emotion regulation, which correlated with improvements “in psychological well-being, subjective health, quality of social relationship, and *work success*” [39] (p. 361, emphasis added).

Another important skill is emotional intelligence, which, in most definitions, also involves emotion regulation. Emotional intelligence (EI, sometimes referred to as EQ) involves the extent to which individuals: (1) recognize their emotions and understand how emotions impact their behavior; (2) control impulsive feelings and successfully manage their emotions; (3) identify others’ emotional cues without letting one’s own emotions interfere with behavior; and (4) maintain good relationships, communicate clearly, influence others, work well in a team, and manage conflict (e.g., [40]). Individuals with higher EI tend to handle stress better than those with lower EI, and EI has been correlated with police performance [41]. Moreover, there have been promising findings that training can improve emotional intelligence (e.g., [42,43,44]) and encouraging efforts at applying this to police officers (e.g., [45]). 

Academy training is crucial to properly prepare recruits to successfully cope with the emotional challenges of police work. In fact, “it may be just as important for a modern day police officer to be emotionally aware as it is for them to be physically fit and knowledgeable about the law” [46] (p. 436). Therefore, it is essential for recruits in the academy to learn how to regulate their emotions in the myriad situations that they are likely to encounter on the job and, more broadly, for the academy experience to increase recruits’ level of emotional intelligence. Integrally linked to this, law enforcement agencies must ensure that academy training teaches recruits evidence-based techniques to successfully manage routine and traumatic stressors.

There has been growing attention to the importance of improving officer wellness, in general, which inherently addresses officers’ emotional regulation and stress tolerance. A recent publication from the U.S. Department of Justice [47] described the various contributors to officers’ poor health outcomes and emphasized that “the organization and its culture contribute to officer health and wellness” (p. 9). This has led to a commitment to provide grants, including one that trains “officers on techniques for self-regulating their emotional and physiological responses to stress” (p. 4).

There are specific activities that police academies can introduce to improve recruits’ wellness and stress tolerance. The first step is to instill a culture of wellness. This begins by structuring the academy to include regular, formal debriefings, which include recruits, veteran officers, and academy staff. The debriefings can be facilitated by a department psychologist, a peer support officer, or a member of the agency’s wellness unit. The debriefings establish a pattern for recruits to talk about their reactions (i.e., thoughts and feelings) about academy performance issues, about incidents about which they heard that occurred on the department, and about any pertinent news reports from anywhere, which involve police officers. These debriefings validate and normalize recruits’ reactions and provide a healthy outlet.

Academies should encourage, if not require, recruits to keep a journal. Starting this practice at the outset of recruits’ careers will launch what has been found to be an important tool to help maintain psychological health. For example, in one study, nurses who wrote freely in a journal each day had less compassion fatigue and more compassion satisfaction than those who did not keep a journal [48]. If academies establish this as a mandatory activity, recruits would not have to share the content of their journals, but would be required to verify that they write each day. The hope is that, once the habit is established, the recruits will continue the practice of journaling (even electronically, e.g., on a computer or smartphone) throughout their careers.

Fundamental to successful emotional regulation and stress tolerance is learning performance enhancement techniques, which keep recruits operating at peak performance levels. Much like athletes who have to learn to control the intensity of emotions in order to compete at their highest level, police officers need to understand the role that their emotions can play in the performance of their duties. The police academy is where this training should begin. In addition to the previously mentioned development of emotional intelligence skills, recruits should be taught skills to reduce acute levels of anxiety (e.g., breathing, mindfulness), which, if not controlled, significantly detract from optimal performance. Rather than training rote repetition of, for example, arrest and control techniques, recruits need more time rehearsing these techniques in highly stressful conditions where they can develop confidence and competence in their ability to manage their own level of internal distress. In addition to remaining calm enough to properly escalate contacts according to the use of force continuum, it is imperative for academies to train recruits how to manage their emotions in order to properly deescalate volatile situations. 

Associated with efforts to improve recruits’ ability to successfully manage acute stress, the academy can promote efforts designed to mitigate recruits’ chronic distress levels. This chronic stress leads to many negative health outcomes [49], which contribute to numerous work-related difficulties. For example, one study showed that recruits experienced less stress and reported better mood after participation in a yoga program that was given during the academy [50]. Fitness, nutrition, sleep hygiene, avoidance of self-medication (e.g., caffeine, alcohol), and activities such as yoga should be strongly promoted and modeled by recruits’ mentors during academy training. The goal is for the recruits to establish a commitment to their personal wellness during the academy and to maintain these good health practices throughout their careers. 

## 4. Social Skills

Police work is socially challenging. In addition to the skills necessary to effectively navigate the difficulties often encountered when interacting with members of the community, police officers are faced with significant obstacles coping with the strain that police work places on their friendships and their relationships with loved ones. This pressure is especially difficult for female police officers and, even more so, for married female officers with children [51]. Police officers often struggle to maintain a healthy work-life balance and may bring their work demeanor home with them, which can negatively impact their family members and, especially, their marriages (e.g., [52,53,54]). Similarly, police officers’ spouses and children endure a variety of difficulties, which, in turn, leads to added stress on the officers. (Readers are referred to Kirschman [2018] for an excellent discussion of the stressors experienced by police families [55].) This added stress, then, adversely affects officers’ work performance. For example, after graduating the police academy and beginning patrol work (often on the weekends and on nightshift), many officers begin to hear grumblings from their spouse and children about never being home and about missing important family functions. These complaints can grow into direct pressure on officers to quit their job. Fundamentally, much of the family-related consternation stems from a lack of preparation and support.

The transactional nature and complexity of police-family stress dictates that law enforcement agencies take steps to address the needs of police families. This can be done in several ways. First, mental health resources should be readily available for officers, their spouses, and their children. Second, agencies can do a better job training their officers to prepare for this source of distress. This training should focus both on detection of early warning signs of family stress and on ways to prevent many common sources of it. Third, agencies can directly engage spouses and family members of their officers. This can be done through peer-support programs (e.g., wives auxiliary; [56]), but also should start much earlier in officers’ careers.

An innovative program to address police-family stress and to directly prepare police families for what to expect when their loved one becomes a police officer, which was implemented by a large, metropolitan police agency, takes place during recruits’ academy training. This begins during a pre-academy orientation, which is the recruits’ first day of employment. Along with signing up for medical benefits, an introduction from command staff, and an orientation from the police union, recruits receive a 30-minute wellness orientation. They hear about the resources that are immediately available to the recruits and all of their family members, including access to chaplains, psychological service providers, and staff of the agency’s Wellness Unit. The recruits receive a brochure to share with their loved ones, which contains direct contact information for all services and wellness benefits, including the clear authorization that accessing services does not require any approval from their command staff.

The program continues with a Family Orientation Day for recruits and all their family members to attend on the first Saturday following recruits’ graduation from the academy. The orientation is facilitated by the agency’s Wellness Unit staff and the Psychological Services staff. The morning is for the new graduates only and includes introductions of chaplains, psychologists, wellness staff, the senior officers in attendance, and each newly graduated officer. The officers discuss why they chose the profession and explain their current family makeup. The morning concludes with a few testimonials from senior officers who share stories about significant issues during their career, which impacted their wellness (e.g., officer involved shootings, a personal tragedy or injury). These officers emphasize what they did to overcome obstacles and how they have specifically coped with trauma.

The family members arrive at noon on Orientation Day to have lunch with their officer. After lunch, the new officers meet with the psychologist for 60–90 min while their family members meet with the staff of the agency’s Wellness Unit. The officers are encouraged to seek regular “wellness checks” and to recognize the importance of good communication with their family members. Simultaneously in another room, the family members hear about detecting early warning signs of distress in their officer. They are given information about how to independently access resources without the need to first obtain approval from their loved one; this includes a promise of anonymity should they ever reach out for some support.

The officers and their family members are reunited for the final few hours of Family Orientation Day. First, an overview of available resources is presented by the chaplains, psychologists, Wellness Unit staff, and members of the agency’s Peer Support Team. Next, for about one hour, a married couple discusses “Dynamics of a Law Enforcement Marriage/Family,” which is followed by a question and answer period. Finally, the day ends with an open discussion of: healthy communication patterns; the need to establish ground rules with each other; the toll of shift work; recognizing “red flags;” reinforcing healthy eating and sleeping habits; and, expectations on the officer and the family by the FTO (Field Training Officer) program. It is essential to have the family members’ understanding, support, and active participation in their own and their officers’ ongoing wellness.

Beyond the support for police families, police academies can seamlessly integrate essential social skill training into current academy curricula. From the Peace Officer Psychological Screening Manual, the skills in question are:

*Social Competence* “involves communicating with others in a tactful and respectful manner, and showing sensitivity and concern in one’s daily interactions” [30] (p. 52).

*Teamwork* “involves working effectively with others to accomplish goals, as well as subordinating personal interests for the good of the working group and the organization. It involves establishing and maintaining effective, cooperative working relationships with coworkers, supervisors, clients, representatives of other organizations, and others” [30] (p. 54).

*Assertiveness/Persuasiveness* “involves unhesitatingly taking control of situations in a calm and appropriately assertive manner, even under dangerous or adverse conditions” [30] (p. 70).

The three specific social skills can be taught and strengthened during police academy with little disruption to current academy practices. The traditional authoritarian academy training style does not provide recruits with role models to emulate when their training officers and academy instructors demonstrate a rigid, autocratic leadership style. Instead, the academy staff can foster social competence by treating recruits “in a tactful and respectful manner” and by providing every recruit with an opportunity to practice effective leadership during training. Recruits should be treated in ways in which police agencies expect their officers to interact with members of the community. To learn how to show sensitivity and concern for others, recruits need to experience how it feels to be treated with sensitivity and concern, which in no way detracts from learning how to maintain officer safety. 

When it comes to teamwork and assertiveness, academies can build in a mechanism for peer level interventions. Staff should make it clear that recruits are expected to confront misconduct (e.g., cheating) and a lack of effort by their peers. Beyond supporting each other during fitness activities, it is crucial for the academy to prepare recruits to deal with conflict that is a natural byproduct of group dynamics. This extends to the academy working to minimize recruits’ us-versus-them attitudes vis-à-vis the community by reinforcing an expectation of cooperation with all stakeholders. This is consistent with procedural justice initiatives, which apply to officers’ relations with community members as well as to law enforcement agencies’ relations with their employees (e.g., [47], p. 13). The goal is for recruits to learn to treat others as they would like to be treated, which is a point that was raised, but largely ignored, decades ago, by the authors in [57]. 

Boosting assertiveness can occur in several ways during the academy. This occurs routinely during scenario work. Also, debriefings and self-reflection after every scenario can help recruits gain confidence and improve their respectful command presence. Some recruits will need to increase assertiveness, while others will need to be taught how to exchange an authoritarian, overbearing, or badge-heavy demeanor with an assertive manner. Academies also should provide every recruit with the experience of testifying in mock trials. Mastery of the social skills requires recruits to practice and rehearse; repetition is the key. 

## 5. Moral Skills

Police work is morally challenging. Officers are regularly confronted with moral dilemmas, which can lead to lapses in ethical behavior. (Readers are referred to Blumberg, Papazoglou, and Creighton [2018] for a comprehensive review of the moral risks of police work [30].) The situations are often fairly mundane, like deciding whether to give a motorist either a ticket or a warning or how to respond when offered a gratuity, e.g., a free meal. At the same time, police officers are routinely confronted with far more serious ethical temptations during the daily discharge of their duties, such as bribes from people looking to avoid arrest and easy access to money and other valuables while securing or collecting evidence at crime scenes. Another morally challenging part of police work stems from officers’ commitment to public safety and crime control. This commitment has been described as the noble cause (e.g., [58]). When officers find themselves justifying these honorable ends (protecting society) at the expense of legal means (e.g., breaking rules to catch offenders), they are committing noble cause corruption [59]. 

It would be a mistake for police executives to assume that all new hires possess the moral maturity to successfully navigate the often ambiguous ethical waters of police work. Similarly, it would be wrong to think that officers either have or do not have these moral skills. The fact is that moral skills can be taught and strengthened. Specifically, one fundamental psychological skill that is required of police officers, according to [60], is, *Integrity/Ethics* which “involve[s] maintaining high standards of personal conduct. It consists of attributes such as honesty, impartiality, trustworthiness, and abiding laws, regulations, and procedures” (p. 61).

Building integrity is not as complicated as it may seem. Most recruits enter law enforcement with self-reported integrity scores that are higher than those obtained from college students [59]. The challenge is for academy staff to instill in recruits a life-long commitment to ethical principles even though routine police work is rife with moral risks [61]. Recruits need to be exposed during the academy to these moral risks. However, rather than lecturing them to stay on the virtuous path (or else!), police academies that adopt the adult learning model will confront recruits with moral dilemmas throughout their training. At every turn, recruits should be asked: What would you do? What should you do? After every scenario, recruits should discuss their rationale as to why they chose the course of action that they did. Regular debriefings should confront the possibility of recruits’ unethical decision-making and reinforce an unwavering commitment to maintaining one’s integrity [62,63].

Another factor associated with moral skills is spirituality. Although law enforcement agencies can support their employees’ affiliation with any religion as well as those who are nonbelievers, it is important for police agencies to promote all officers’ commitment to non-religious spiritual practices. On one hand, spirituality can help officers maintain their sense of purpose and meaning, which provides some insulation from the deleterious effects of repeated exposure to human suffering (e.g., [64]). Likewise, spirituality was found to “buffer stress affecting brain connections” in a sample of police officers [65] (p. 241). At the same time, although “spirituality might be associated with a slight reduction in burnout,” other factors, such as officers’ ethnicity and level of family support, also impact stress levels [66] (p. 6). There have been some mixed findings about the specific benefits of spiritual practices among police officers (e.g., [67]). 

Nevertheless, there are many reasons why police academies should encourage recruits to develop their own personalized spiritual program. Resources (e.g., books, Chaplains, peer mentors) should be available to recruits. At several times during the training, recruits should be asked to reflect on their purpose and to discuss why they want to be a police officer. However, regardless of the possible health benefits and restorative effects of spiritual practices for police officers’ wellness, it also was found that ethical decision-making in the workplace is related to employee spirituality (e.g., [68]). Therefore, to facilitate the training of ethical police officers, academies should consider ways to bolster recruits’ spirituality.

## 6. Conclusions

Contemporary policing requires contemporary police training, which incorporates andrological teaching principles. When academies shift from an authoritarian, paramilitary style to an adult learning model, recruits can develop and strengthen important psychological skills that are essential for today’s effective police officers. However, this shift requires two significant initiatives if it is to be successful. First, academy instructors, training officers, and mentors need to support and believe in the value of this change. All of the trainers, also, need to be thoroughly trained to be able to properly train recruits with the aforementioned psychological skills. Academies can establish their own methods of standardized assessment for each skill, ensure satisfactory performance of each skill, provide opportunities for remediation, and require that minimum standards of each skill are met in order for recruits to graduate.

The second important factor required for psychological skills training to become endemic in police academy training is a culture shift at the organizational level. Police agencies must see the value of a psychologically skilled officer and demand that training academies emphasize development of these skills in their police recruits. In doing so, police executives will guide academies to focus less on testing recruits and more on assessment, which measures if recruits are able to apply what they are learning. Typically, when it comes to the application of what is learned in the academy, too much is left to FTOs when much more could be done during the academy, especially when it comes to demonstrating minimum standards of the psychological skills. In other words, police agencies should draw a clear line in the sand that no recruits will be sent to field training before they have satisfactorily demonstrated these important psychological skills.

This raises another important issue. There should be greater continuity between recruits’ academy training and their field training. To accomplish this, academy staff and the FTO program staff need to do a better job collaborating with each other. This alliance shows recruits that there is not some arbitrary distinction between what is expected from them in the academy and the behavioral standards once they graduate and begin working on the streets. For that matter, along with other skills initially taught during the academy (e.g., firearms, defensive tactics, and emergency vehicle operation), police executives should view psychological skills as perishable and begin to require ongoing training and requalification of these skills throughout officers’ careers. The importance of demonstrating proficiency of one’s psychological skills should extend beyond the training academy.

Finally, given the time limitations of police academy training and the mandated content that recruits must learn, some may contend that there is not enough time to focus on building recruits’ psychological skills. We argue that some innovation is warranted, as the psychological skills are as important as any other facet of police work. One solution is for police agencies to develop criteria for basic certification in certain police knowledge and skills (e.g., firearms proficiency, radio codes, law, penal code, and emergency driving), which applicants would have to achieve on their own prior to being hired. This parallels what many fire departments have established; applicants will only be considered for employment as a firefighter after they have acquired paramedic certification on their own. In this way, with a basic law enforcement certificate achieved on their own prior to employment, recruits have proven their commitment to the profession. Then, police academy training can focus on application of those skills, development of higher-order police techniques, and strengthening of the psychological skills.

Short of the requirement for police applicants to independently attain some form of certification prior to employment, police agencies can do a better job preparing new hires prior to the start of the academy. There is generally quite a bit of time between the beginning of the hiring process and candidates’ academy start date. Many jurisdictions already offer pre-academy physical training whereby candidates train and are exposed to the academy’s fitness expectations. This is where the acculturation process begins. Similarly, there should be pre-academy psychological “work-outs.” Candidates could learn about and have an opportunity to practice in areas such as procedural justice, community relations, stress management, and the various psychological skills. It is vital for police executives to find opportunities for their recruits and officers to learn, practice, and demonstrate the psychological skills that are critical for successful police performance.
